# Two-dimensional semiconductors survive and thrive in outer space

**DOI:** 10.1093/nsr/nwaf181

**Published:** 2025-05-09

**Authors:** Junqiao Wu

**Affiliations:** Department of Materials Science and Engineering, University of California, Berkeley, USA

Outer space is the vast expanse that exists beyond Earth's atmosphere, stretching between celestial bodies. It contains extremely low particle densities, forming a near-perfect vacuum composed primarily of hydrogen and helium plasma. This space is permeated by electromagnetic radiation, cosmic rays, neutrinos, magnetic fields and interstellar dust. The surface of space objects, if not thermally managed, would also experience a wide temperature swing. Survival of electronics in such harsh environments in outer space is critical for space exploration. Continuous exposure to ionizing radiation, such as from the Van Allen belts, can cause gradual degradation of Si-based electronic components, leading to increased leakage currents, threshold shifts and reduced functionality. To enhance their endurance, various techniques like radiation-hardened designs, shielding and the use of alternative radiation-hard materials like silicon carbide (SiC) are employed [1].

Understanding the physical behavior of electronic materials, such as semiconductors [2], thermoelectrics [3] and ferroelectrics [4], under radiation exposure typical of outer space has been an active and important research field. For example, the electrical conductivity of a semiconductor may either increase (as in InN or CdO) or decrease (as in Si or GaAs) depending on the unique physics of point defect generation, ionization and charge trapping in the material [5].

2D semiconductors, particularly transition metal dichalcogenides, have emerged as promising materials with regard to continuing the miniaturization of microelectronic devices. Naturally, an important question arises: how robust are monolayer 2D semiconductors when subjected to the harsh conditions of outer space? A recent work [6] by Dr. Ruitao Lv and colleagues offers a preliminary answer: these materials are electronically quite stable in space conditions, with superior resistance in their optical properties against radiation damage.

**Figure 1.**
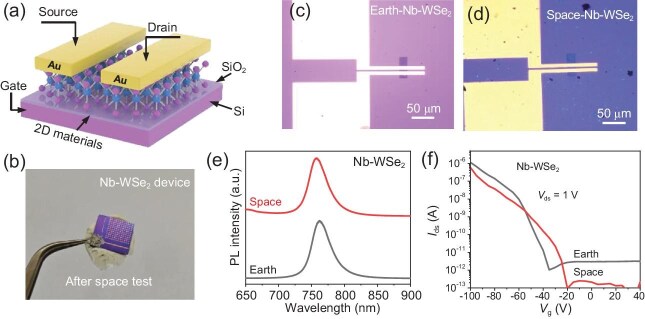
(a) Schematic diagram and (b) photograph of a field-effect-transistor (FET) device made of Nb-doped WSe_2_ after the space test. (c and d) Optical microscope images of the FET device before and after the space test. (e) Photoluminescence of a Nb-doped WSe_2_ monolayer, and (f) source-drain current as a function of gate voltage of the transistor before and after the space test. Adapted with permission from Ref. [6].

Using the recoverable satellite Shijian-19, the team tested the optical properties and field-effect-transistor (FET) performance of WSe₂ and Nb-doped WSe₂ after a 14-day orbit in which they experienced radiation in outer space. For comparison, they also measured leakage currents in a SiO₂ dielectric layer (∼300 nm thick). Despite an expected two-order-of-magnitude increase in leakage current in SiO₂, Raman and photoluminescence measurements of the 2D semiconductors showed negligible degradation (Fig. 1). Furthermore, FETs made from these 2D materials maintained a high ON/OFF ratio (∼10⁶) after the flight, though some gate threshold voltage shifts were observed, likely due to accumulated interface charge traps or surface contamination.

This pioneering work highlights 2D semiconductors as a promising material platform for light emitters, transistors, solar cells and sensors suited for space exploration and other extreme environments. It would be exciting to see following-up work that quantifies the radiation hardness of 2D materials benchmarked against other conventional semiconductors such as Si in space environment.
